# Unilateral Proptosis Mimicking an Eye Tumour: A Case Report

**DOI:** 10.7759/cureus.98229

**Published:** 2025-12-01

**Authors:** Ana Rita G Magalhães, Ana M Carvalho, Pedro Almeida, Diana Mimoso, Sofia Perdigão, Beatriz T Exposito

**Affiliations:** 1 Internal Medicine, Unidade Local de Saúde de Trás-os-Montes e Alto Douro, Chaves, PRT; 2 Internal Medicine, Centro Hospitalar de Trás-os-Montes e Alto Douro, Chaves, PRT

**Keywords:** exophthalmos, graves-basedow, orbitopathy, thyroid eye disease, tumor, unilateral

## Abstract

We report the case of a 56-year-old woman who presented to the Emergency Department with unilateral exophthalmos of the left eye, accompanied by recurrent bitemporal headache. Her past medical history was significant for hypertension, ischemic heart disease, anxiety, depression, and obesity. On clinical examination, the patient was hemodynamically stable, alert, and fully oriented. A brief neurological assessment revealed no focal deficits.

Initial laboratory investigations, including complete blood count, serum electrolytes, and coagulation profile, were within normal limits. D-dimer levels were negative. A contrast-enhanced cranial computed tomography (CT) scan ruled out both intracranial space-occupying lesions and cerebral venous sinus thrombosis. Orbital magnetic resonance imaging (MRI) excluded any primary orbital pathology.

Endocrine evaluation of the hypothalamic-pituitary-thyroid axis revealed a suppressed thyroid-stimulating hormone (TSH) level of 0.010 mUI/L (reference range: 0.27-4.20 mUI/L) and a free T4 level of 21.59 pmol/L (reference range, adjusted for age and sex: 8.24-21.0 pmol/L). Given the suspicion of Graves-Basedow disease with atypical unilateral ocular involvement, an autoimmune panel was performed. TSH receptor antibodies (TRAb) were markedly elevated at 11.0 U/L (reference range: 0-1.8 U/L), confirming the diagnosis of Graves’ disease.

Graves’ disease is an autoimmune thyroid disorder that typically presents with bilateral exophthalmos due to inflammatory changes and expansion of the extraocular muscles and orbital adipose tissue. However, unilateral exophthalmos, although uncommon, can occur as a manifestation of Graves’ orbitopathy.

## Introduction

Graves' disease is a tissue-specific autoimmune disease characterized by the production of autoantibodies that stimulate the thyrotropin (thyroid-stimulating hormone, or TSH) receptor on thyroid follicular cells. This pathological stimulation leads to the unregulated synthesis and production of thyroid hormones, thyroxine (T4) and triiodothyronine (T3) [1].

The most common extrathyroidal manifestation of this disease involves the eye and is known as thyroid eye disease (TED) or Graves' orbitopathy [2]. In the European population, the prevalence is approximately 10/10,000 individuals [3], with 30.9% for asymmetric and 10.7% for unilateral disease, and approximately 75% of patients with thyrotoxicosis develop TED within one year of diagnosis [4].

Usually, the disease presents bilaterally and symmetrically, with symptoms such as lid retraction, proptosis, and diplopia [3]. Unilateral and asymmetrical Graves' disease poses a significant diagnostic challenge due to its resemblance to a range of other conditions, including orbital tumour/pseudotumour, orbital cellulitis, cavernous sinus thrombosis, and intra-orbital neoplasms [3-5].

We present a clinical case demonstrating the diagnostic work-up of unilateral exophthalmos with TED as the underlying cause.

## Case presentation

We report the case of a 56-year-old woman who presented to the Emergency Department with unilateral exophthalmos of the left eye, accompanied by severe holocranial headache and episodes of vomiting. She did not report photophobia or phonophobia. Her medical history included hypertension, ischemic heart disease, anxiety, depression, and obesity.

**Figure 1:**
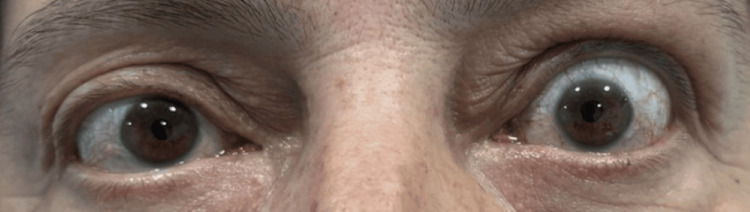
Exophthalmos of the left eye with conjunctival redness and lid retraction

Upon clinical examination, the patient was hemodynamically stable, alert, and fully oriented. Neurological examination revealed no focal deficits, impairment of higher cortical functions, or diplopia. Notably, proptosis with retraction of the upper eyelid in the left eye was observed (Figure 1). The right eye was deemed normal in appearance by the patient and clinicians, and old photographs were reviewed for comparison.

Initial laboratory investigations - including a complete blood count, serum electrolytes, and coagulation profile - were within normal limits, with negative D-dimer levels. A contrast-enhanced cranial computed tomography (CT) scan excluded intracranial space-occupying lesions and cerebral venous sinus thrombosis.

**Figure 2:**
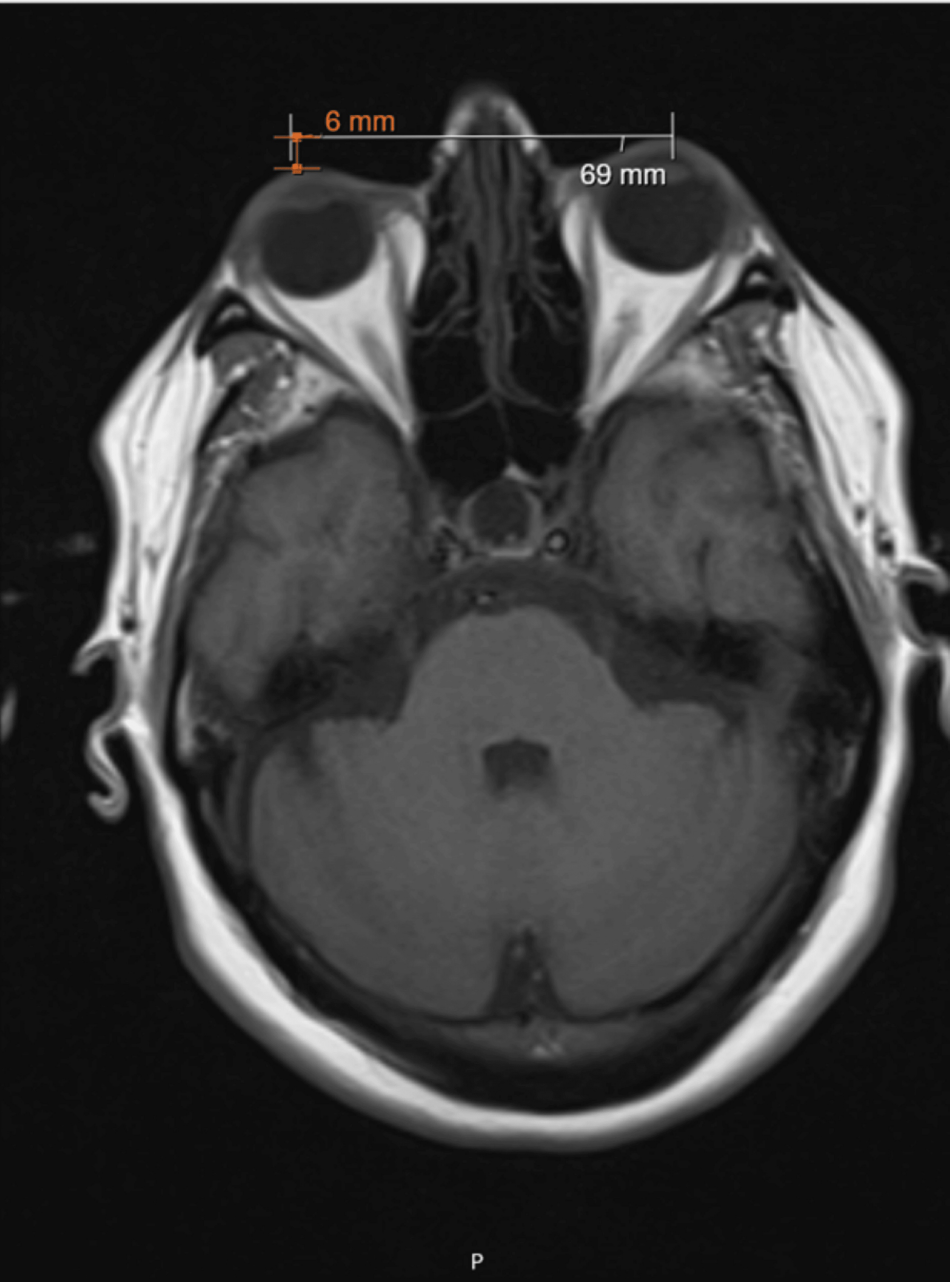
Orbital MRI (T1-weighted sequence) This image demonstrates a 6 mm asymmetry with anterior displacement of the left globe, consistent with unilateral proptosis. MRI, magnetic resonance imaging

Subsequent orbital magnetic resonance imaging (MRI) revealed no primary orbital pathology or space-occupying lesions, but raised suspicion of Graves' disease. An asymmetry of the orbits was noted, with the left orbit protruding 6 mm further than the right (Figure 2).

**Figure 3:**
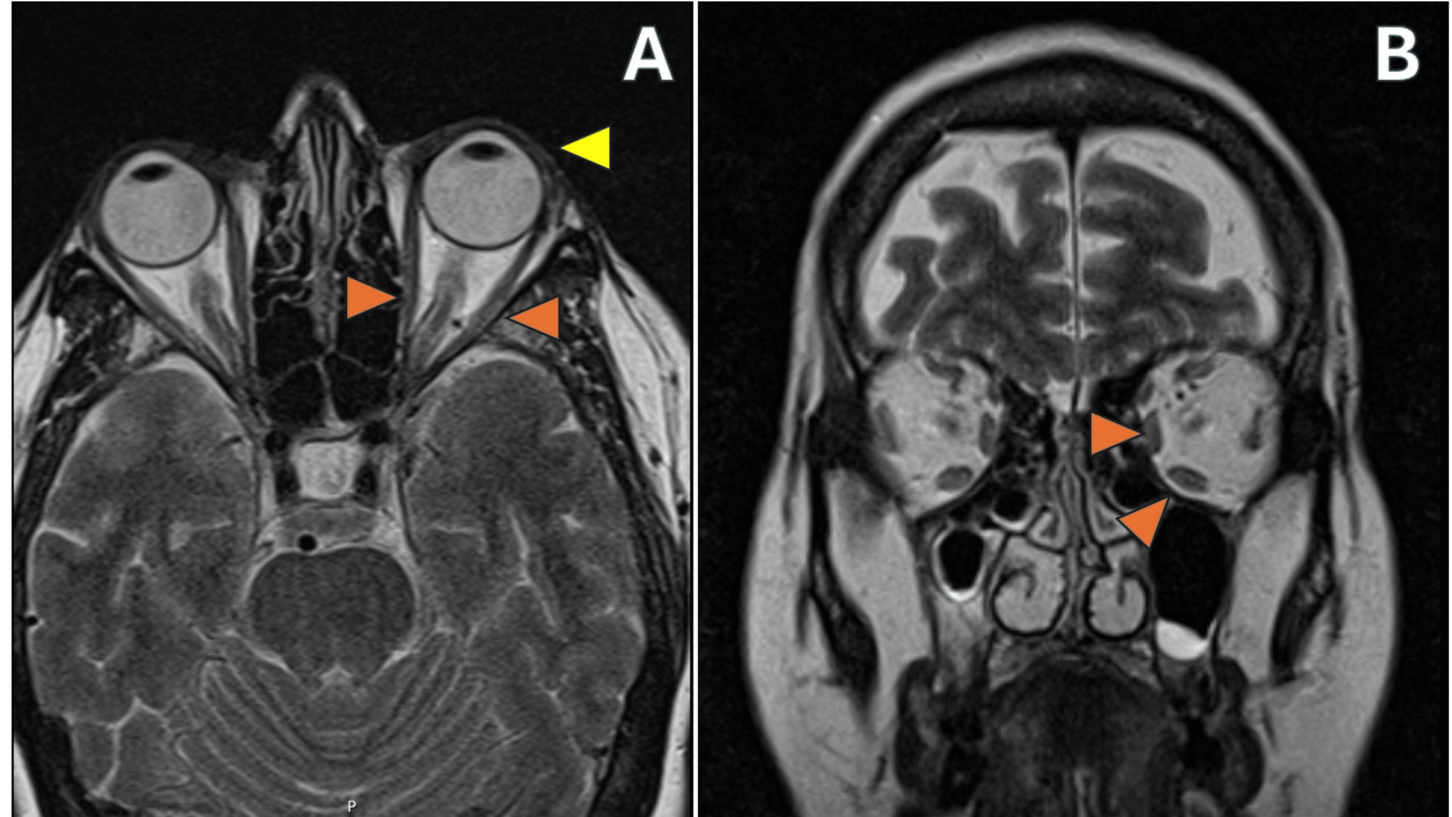
Orbital MRIs Panel A (axial T2-weighted sequence): Mild inflammation of the extraocular muscles can be observed, characterized by increased signal intensity in the muscle mass, sparing the tendon, highlighted by orange arrows. Anterior protrusion of the left globe is also noted, indicated by the yellow arrow. Panel B (coronal T2-weighted sequence): Hypertrophy of the left extraocular muscles, as indicated by the orange arrows. MRI, magnetic resonance imaging

**Figure 4:**
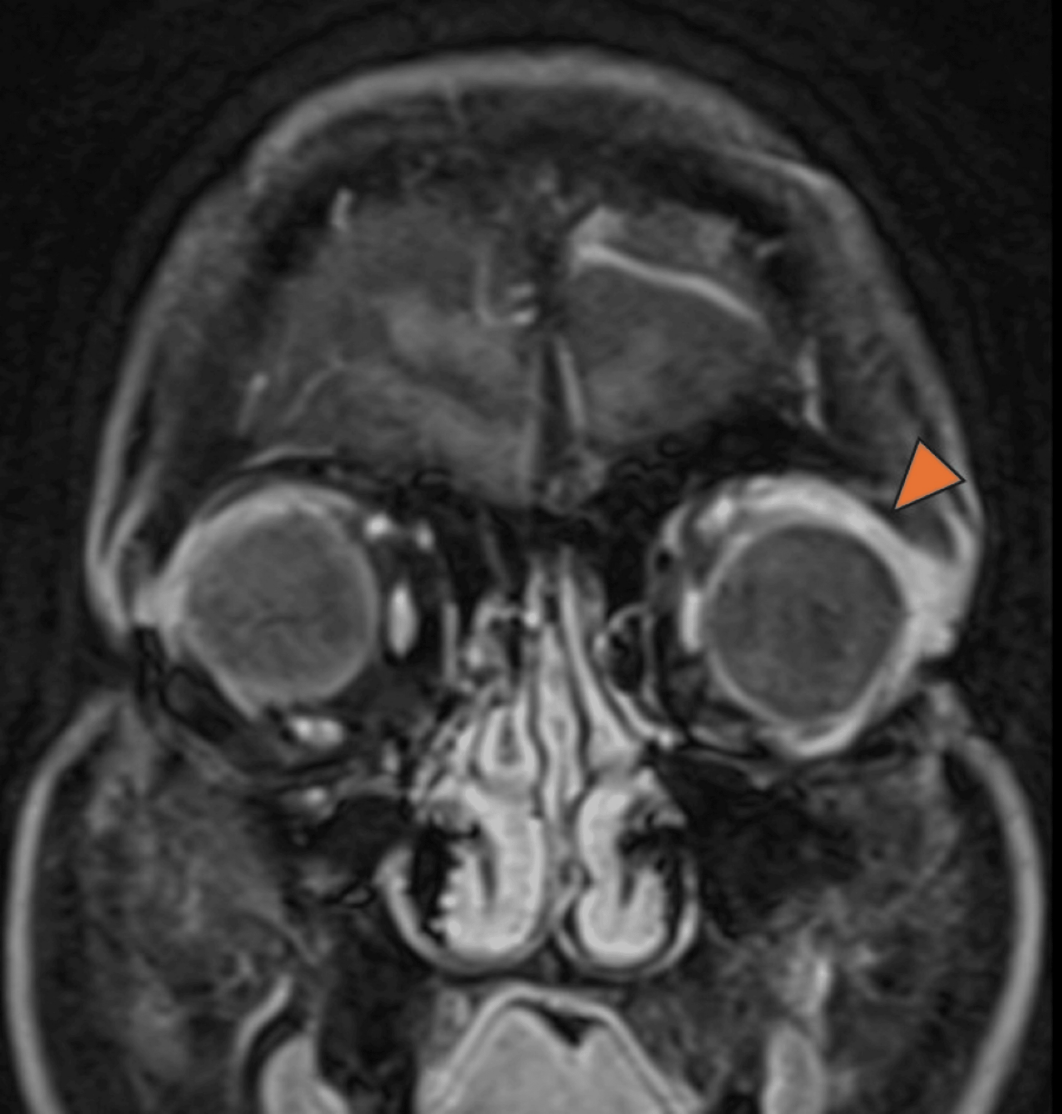
Orbital MRI (coronal T1-weighted sequence) Inflammation of the left lacrimal gland is observed, evidenced by increased signal intensity (hyperintensity) as indicated by the orange arrow. MRI, magnetic resonance imaging

On the T2-weighted imaging, mild inflammation of the extraocular muscles in the left eye (Figure 3A, orange arrows) is observed, as noted by hyperintensity of the muscle belly with tendon sparing. At the same time, coronal images exhibited mild hypertrophy of the extraocular muscles (Figure 3B, orange arrows). Additionally, T1-weighted coronal images showed inflammation of the left lacrimal gland (Figure 4). 

These radiological findings prompted an evaluation of the hypothalamic-pituitary-thyroid axis, revealing a suppressed TSH level of 0.010 mUI/L (reference range: 0.27-4.20 mUI/L) and an elevated free T4 level of 21.59 pmol/L (reference range: 8.24-21.0 pmol/L). Considering the suspicion of Graves' disease with atypical unilateral ocular involvement, an autoimmune panel was conducted, revealing markedly elevated TSH receptor antibodies (TRAb) at 11.0 U/L (reference range: 0-1.8 U/L), confirming the diagnosis of Graves' disease with orbitopathy (Table 1).

**Table 1:** Thyroid function laboratory results Summary of biochemical findings relevant to thyroid status, including hormone levels and autoantibody profiles, consistent with Graves’ disease.

Laboratory parameter	Result	Normal range
Thyroid-stimulating hormone (TSH)	0.010 mUI/L	0.27-4.20 mUI/L
Free T4	21.59 pmol/L	8.24-21.0 pmol/L
TSH receptor antibodies (TRAb)	11.0 U/L	0-1.8 U/L

## Discussion

TED, also known as Graves' orbitopathy or Graves' ophthalmopathy, is the most common extrathyroidal manifestation of Graves' disease [3,4]. It is present in 25%-50% of Graves' patients, and nearly 75% of patients with thyrotoxicosis develop TED within the first year of diagnosis [4].

TED is an autoimmune inflammatory orbital disease. The proliferation of orbital fibroblasts, increased adipogenesis, and extracellular matrix expansion are involved in its pathophysiology, and TRAbs are a key determinant. This triggers a series of interconnected processes. Proinflammatory cytokines are released, prompting orbital fibroblasts to multiply and transform into myofibroblasts and adipocytes. At the same time, signalling through the TSHR/IGF-1R complex drives the production of hyaluronic acid and glycosaminoglycans, which contribute to tissue expansion. These molecular changes, combined with the recruitment and activation of T and B lymphocytes, amplify inflammation and lead to muscle swelling, increased orbital fat, and progressive tissue remodelling [6]. The TRAb titre, active smoking, duration of thyroid dysfunction, and clinical activity score (CAS; see Table 1) at baseline are considered the main risk factors for developing TED [3,7]. TED most often presents bilaterally and symmetrically, with lid retraction, exophthalmos, and diplopia, but may present asymmetrically or even unilaterally in a small minority of patients [3,6,8]. This condition can be sight-threatening [6].

The patient in this case was a female, a non-smoker without a previous history of thyroid disease. Her laboratory results revealed a suppressed TSH (0.010 mUI/L), slightly elevated free T4 (21.59 pmol/L), and markedly elevated TRAb (11.0 U/L), even though the patient did not present any other common symptoms of thyrotoxicosis. Based on clinical features and symptoms, a CAS of 4 points can be calculated, indicating active TED (Table 2). The CAS was not calculated for the right eye, since it was deemed normal. 

**Table 2:** Clinical activity score (CAS) CAS < 3 = inactive thyroid eye disease (TED); CAS ≥ 3 = active TED. Table credit: Adapted from the 2021 European Group on Graves' Orbitopathy (EUGOGO) guidelines [7].

Assessment of activity	Points if present
Spontaneous retrobulbar pain	1
Pain on attempted upward or downward gaze	1
Redness of the eyelids	1
Redness of the conjunctiva	1
Swelling of the caruncle or plica	1
Swelling of the eyelid	1
Chemosis/Swelling of the conjunctiva	1

TED is an autoimmune condition that primarily affects the extraocular muscles and lacrimal glands, leading to inflammation and, over time, fibrosis [3,4,8,9]. While most patients present with bilateral involvement, a smaller but clinically significant group presents with unilateral or markedly asymmetric disease [1,5,8]. This atypical pattern often complicates diagnosis because unilateral proptosis is more commonly linked to orbital tumours or inflammatory conditions. Importantly, the degree of asymmetry does not always reflect the severity of thyroid dysfunction; instead, it may be driven by localized immune activity or anatomical differences [2,3].

Imaging is essential for accurate diagnosis. MRI offers excellent soft-tissue detail and can reveal muscle oedema, a sign of active inflammation. Classic TED findings include enlargement of the muscle belly with tendon sparing, expansion of orbital fat, and, sometimes, lacrimal gland enlargement [6,9]. These features help distinguish TED from other conditions: orbital pseudotumour typically involves both muscle and tendon, cellulitis shows diffuse fat stranding, and neoplasms appear as discrete masses [6]. Interestingly, recent studies have shown that lacrimal gland enlargement, once thought rare, can occur in both bilateral and unilateral TED, adding another layer of complexity to the differential diagnosis [9].

The clinical impact of asymmetry goes beyond diagnostic challenges. Patients with unilateral TED often face delays in treatment because the presentation mimics other orbital diseases [1,4]. From a psychosocial perspective, asymmetry tends to cause more distress than bilateral disease, as the cosmetic imbalance is more noticeable and can significantly affect self-image and confidence [2,5]. Functionally, severe asymmetry may lead to diplopia due to uneven muscle involvement, and, in rare cases, compressive optic neuropathy can develop on the more affected side [3,8]. Surgical management is also more demanding: orbital decompression often needs to be customised or staged to restore symmetry, and strabismus correction is complicated by uneven muscle involvement, increasing the risk of postoperative diplopia [2,4]. Moreover, asymmetric disease may signal a more aggressive local immune process, making early intervention crucial [9,10]. Close follow-up is recommended, as up to 20%-30% of patients progress to bilateral involvement within two years [5,8].

Treatment strategies for unilateral TED generally follow those for bilateral disease. Anti-thyroid drugs remain the cornerstone for managing underlying Graves’ disease [10], while mild cases benefit from local measures such as lubricants and selenium supplementation [6,10]. For moderate to severe disease, systemic corticosteroids - either oral or intravenous - are still first-line therapy [8]. However, the introduction of targeted biologics has changed the landscape. Teprotumumab, an IGF-1R inhibitor, has shown remarkable efficacy in reducing proptosis and improving motility, even in asymmetric cases, potentially reducing the need for complex surgical interventions [9,10]. Acting early is key to preventing irreversible fibrosis and persistent asymmetry [2,5].

## Conclusions

In conclusion, TED is predominantly characterized by bilateral manifestations; however, healthcare professionals must maintain a high level of vigilance for atypical presentations, such as unilateral exophthalmos. The early identification of TED in these instances is paramount to prevent misdiagnosis and the subsequent delay in therapeutic intervention, which can result in irreversible ocular complications. The unilateral presentation of exophthalmos may obscure the systemic autoimmune aetiology, particularly in euthyroid individuals, thereby necessitating a thorough diagnostic workup and a high index of suspicion. By incorporating TED into the differential diagnosis of unilateral exophthalmos, clinicians can facilitate prompt management, safeguard visual function, and ultimately enhance patient outcomes.
